# Supplementary figures and images for: Microbial Community and Metabolome Analysis of the Porcine Intestinal Damage Model Induced by the IPEC-J2 Cell Culture-Adapted Porcine Deltacoronavirus (PDCoV) Infection
Source: Microorganisms. 2024 Apr 27;12(5):874. doi: 10.3390/microorganisms12050874 (PMC11124095; doi:10.3390/microorganisms12050874)

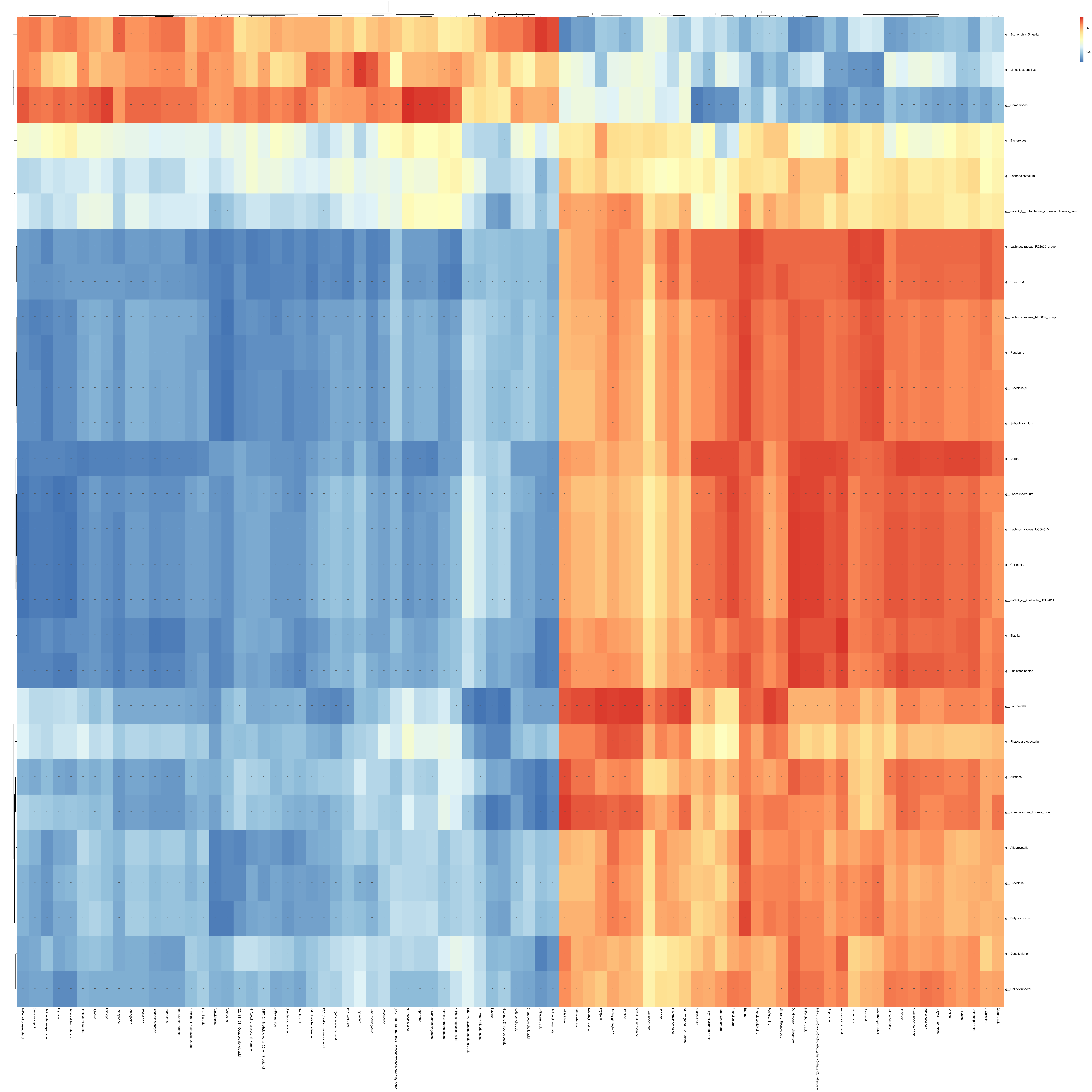

Supplement: Supplementary file 1 [file microorganisms-12-00874-s001.zip › Figure S1.pdf]
